# Weight-based disparities in perinatal care: quantitative findings of respect, autonomy, mistreatment, and body mass index in a national Canadian survey

**DOI:** 10.1186/s12884-024-06928-8

**Published:** 2024-11-08

**Authors:** Nisha Malhotra, Cecilia M. Jevitt, Kathrin Stoll, Wanda Phillips-Beck, Saraswathi Vedam, the RESPCCT Study Team

**Affiliations:** 1 Department of Family Practice, Faculty of Medicine, University of British Columbia, Vancouver, Canada; 2 University of Manitoba, Indigenous Research Chair in Nursing, First Nations Health and Social Secretariat of Manitoba, Manitoba, Canada

**Keywords:** Obesity, Weight bias, Weight stigma, High body mass index, Mistreatment in perinatal care, Patient-reported experience, PREMs, Healthcare disparities

## Abstract

**Background:**

Qualitative studies document episodes of weight-related disrespectful care, particularly for people with high body mass index (BMI ≥ 30) and reveal implicit and explicit biases in health care providers. No large quantitative studies document the pervasiveness of weight stigma or if experiences change with increasing BMI.

**Methods:**

The multi-stakeholder RESPCCT study team designed and distributed a cross-sectional survey on the experiences of perinatal services in all provinces and territories in Canada. From July 2020 to August 2021, participants who had a pregnancy within ten years responded to closed and open-ended questions. Chi square analysis assessed differences in mean scores derived from three patient-reported experience measures of autonomy (MADM), respect (MOR), and mistreatment (MIST). Controlling for socio-demographic factors, multivariate logistic regression analysis explored relationships between different BMI categories and respectful care.

**Results:**

Of 4,815 Canadians who participated, 3,280 with a BMI of ≥ 18.5 completed all the questions. Pre-pregnancy BMI was significantly associated with race/ethnicity, income sufficiency, and education but not with age. Individuals with higher BMIs were more likely to experience income insufficiency, have lower levels of education, and more frequently self-identified as Indigenous or White. Those with BMI ≥ 35 exhibited notably higher odds of reduced autonomy (MADM) scores, with an unadjusted odds ratio of 1.62 and an adjusted odds ratio of 1.45 compared to individuals with a normal weight. Individuals with BMIs of 25–25.9, 30–34.9, and ≥ 35 exhibited odds of falling into the lower tercile of respect (MOR) scores of 1.34, 1.51, and 2.04, respectively (*p* < .01). The odds of reporting higher rates of mistreatment (top 33% MIST scores) increased as BMI increased.

**Conclusions:**

While socio-demographic factors like race and income play significant roles in influencing perinatal care experiences, BMI remains a critical determinant even after accounting for these variables. This study reveals pronounced disparities in the provision of respectful perinatal care to pregnant individuals with higher BMIs in Canada. Data suggest that those with higher BMIs face disrespect, discrimination, and mistreatment. Identification of implicit and explicit weight bias may give providers insight enabling them to provide more respectful care.

## Introduction

Individuals with high weights cannot hide from weight-based stigma. Like variations in skin colour, weight is a physical attribute that is visible and often judged before the person becomes known. Weight-based stigma, also known as weight bias, includes implicit and explicit biases [1, 2]. Implicit biases are automatically triggered negative attitudes, beliefs and judgements that happen without conscious awareness. A person, on the other hand, can recognize an explicit bias. Both implicit and explicit biases can lead to inaccurate and negative views of a person’s character and abilities, sometimes producing unequal and discriminatory behavior [1–3]. Historical social bias against individuals with high weights is present in Europe, North America, and Australia [4–7]. This bias increased as rates of obesity as measured by body mass index (BMI, weight kg/m^2^ height) doubled and tripled in many countries over the last 50 years [8]. The increased reports of obesity led to specialized bariatric care guidelines and fueled industries aimed at weight loss through exercise, special foods, dietary supplements, and weight-reducing surgeries and medications [9–13]. The weight-centric risk and pathology approach to medical management and therapies instead of a health-based focus can increase microaggressions and mismanagement that are experienced as weight stigma by pregnant people [14, 15]. Some researchers find weight discrimination more prevalent than discrimination based on race or ethnicity [16].

In reality, pregnant people may experience the intersecting stigmas of being female, racialized, gender-fluid, and of high weight [17, 18]. For example, there are three distinct Indigenous groups in Canada—First Nations, Inuit (Inuk), and Métis—each with their own rich cultures, languages, and histories [19]. Indigenous peoples in Canada have had unique experiences and face systemic barriers and challenges compared to the other racialized groups from land displacement, and water pollution, starvation and food experimentation being done on Indigenous children in residential schools [19–23]. Policies such as the forced evacuation of pregnant women from rural and remote communities disrupt their support systems during childbirth, which highlight the ongoing impacts of colonization [24].

Internalized weight stigma stemming from experiencing body shaming reduces the uptake of health care by women [25–27]. Women with greater body-related guilt report healthcare stress fearing further mistreatment by healthcare providers, which ultimately leads to healthcare avoidance [25]. Pregnant people do want guidance about nutrition and weight gain during pregnancy; however, they do not want their actions to be prejudged or stereotyped, and most of all, they want to feel respected [7, 28].

Weight bias is not limited to the public. It is documented in studies of health care providers of all kinds: nurses, midwives, and physicians [1, 2, 5, 29–31]. A recent survey of certified nurse-midwives and certified midwives in the United States found that more than 70% had some level of implicit bias, although bias levels were lower than published studies of other health care providers and the public [1]. Midwives, who themselves had lower BMIs, had higher levels of implicit and explicit weight bias. Younger midwives had lower levels of implicit weight bias, while midwives who identified as Black had higher levels of explicit bias [2].

Although weight stigma has been documented in qualitative studies and surveys, no large quantitative study has documented the overall pervasiveness of weight stigma during pregnancy by weight categories. In addition, none has applied person-centred metrics by population characteristics in a quantitative survey of respectful maternity care. Using three validated patient-reported experience measures (PREMs), this study aimed to document the impact of weight stigma during pregnancy in a large national sample and determine if experiences of weight stigma increased with increasing weight as measured by body mass index (BMI).

## Methods

The RESPCCT (Research Examining Stories of Pregnancy and Childbearing in Canada Today) study is a participatory action research project that was conceived by a group of reproductive health researchers, Indigenous scholars, community members and clinicians convened by the Birth Place Lab (University of British Columbia). The primary aim of the study was to understand the experiences of perinatal service users in Canada, specifically experiences of respectful care along with its opposites: mistreatment, discrimination and disrespect. The Behavioral Research Ethics Board at the University of British Columbia granted approval to the study (#H18-01961) in May 2020 and renewed approval for the participatory analysis phases in 2024. A Community Steering Council was convened to ensure community priorities were centered during the project. Since this study captured some Indigenous specific data, Indigenous community members were included on the Council to ensure those data were appropriately reviewed and represented. The RESPCCT study also aimed to document perinatal experiences across Canada among different groups of childbearing people with a focus on populations that are underrepresented. This secondary analysis focused on the treatment of pregnant people with high weights as measured by body mass index.

### Survey development

First, the study team conducted a systematic review of the literature and subsequent Delphi process to identify indicators of respectful maternity care (RMC) for inclusion on the RESPCCT survey. During the systematic review the team identified 310 potential RMC indicators, which were reduced to 201 by the study team after removing items that did not measure RMC, were unclear or redundant [32]. Next, the set of 201 items was then further reduced to 156 via two rounds of surveys with experts and community members with lived experience of receiving perinatal care. These items were then presented to the community steering council in collaboration with the study team, to further refine items and add new items that were not captured by the Delphi process but resonated with their experience of receiving perinatal care. The final RESPCCT survey included 388 items overall, with 210 that were relevant to respectful maternity care. The items covered 17 domains of RMC [33]. The survey was offered in 8 languages.

### Recruitment

To ensure data collection across multiple regions and populations, 18 Regional Recruitment Community Coordinators were strategically deployed, to spread information about the study through their networks. Several of the RCCs identified with communities that are historically underrepresented in perinatal research, including people with disabilities, racialized childbearing people and those from sexual and gender minority groups. The RESPCCT team developed advertisements for social media in several different languages, using a wide range of images of pregnant people, to ensure that potential participants could see themselves represented. The team also worked with many non-governmental organization partners to enhance participation of underrepresented service users and encouraged snowball sampling, for example, participants forwarding the study ads to other eligible people within their networks. Details on item generation, survey construction, and recruitment are published elsewhere [33].

### Measures

The current analysis included two validated patient-reported experience measures (PREMS) and one adapted PREM. The patient experience measures used in the RESPCCT survey are: 1) *Mothers Autonomy in Decision Making* (MADM) scale, a 7-item scale that rates the degree to which health care providers facilitated autonomy in decision-making when discussing options for care (Likert-type, range of scores 7–42) [34]; and 2) *Mothers on Respect* (MOR*)* index, a 7-item Likert scale that assesses comfort with asking questions, accepting or declining options for care; and/or degree of coercion or cultural respect (range 7–42) [35]. One measure was adapted: the; and 3) *Mistreatment* (MIST-15) index: an expanded version of a previously validated 7-item index that assesses different types of mistreatment and disrespectful behavior (Table 1) [36]. Respondents noted the type(s) of health care provider (HCP) they were reporting on (Family Physician, Midwife, Obstetrician, Nurse or Other) and had the option of choosing ‘Not applicable’. Each time a respondent checked one or more HCP types and did not check NA, they received a score of 1, whereas those who checked NA received a score of 0. The 15 items then were summed.

**Table 1:** Mothers Autonomy in Decision Making (MADM), Mothers on Respect (MOR), and Mistreatment (MIST) Items

**1. MADM: The seven item autonomy scale**
1.My provider asked me how involved in decision making I wanted to be
2.My provider told me that there are different options for my maternity care
3.My provider explained the advantages/disadvantages of the maternity care options
4.My provider helped me understand all the information
5.I was given enough time to thoroughly consider the different care options
6.I was able to choose what I considered to be the best care options
7.My provider respected my choices
**2. MOR-7 Index: The seven questions used to assess respect**
1.I felt comfortable asking questions
2.I felt comfortable declining care that was offered
3.I felt comfortable accepting the options for care that my doctor or midwife recommended
4.I felt pushed into accepting the options my doctor or midwife suggested
5.I chose the care options that I received
6.My personal preferences were respected
7.My cultural preferences were respected
**3. MIST Index: The 15 questions used to assess Mistreatment**
During my labour and/or birth, I experienced the following interactions with one or more providers:
1.My physical privacy was violated (e.g. being uncovered or having people in the delivery room without my consent)
2.My health care provider(s) shouted at or scolded me
3.My health care provider(s) withheld treatment or forced me to accept treatment that I did not want
4.My health care provider(s) threatened me
5.Health care provider(s) ignored me, refused my requests for help, or failed to respond to myrequests for help in a reasonable amount of time
6.I experienced physical abuse (including aggressive physical contact, refusal to provide anesthesia for an episiotomy, etc.)
7.My healthcare provider(s) talked about me as if I was not there
8.My healthcare provider(s) walked in and looked at my chart without speaking to me first
9.I was discouraged from engaging in cultural, traditional, or religious practices
10.My health care provider(s) or other staff member(s) made negative comments to me regarding my sexual activity
11.My health care provider(s) or other staff member(s) made negative comments about my physical appearance (such as my weight, private parts, cleanliness, or other parts of my body)
12.I was mocked by my health care provider(s) or other staff
13.During my childbirth I felt neglected by my health care provider(s)
14.I was left unattended by my health care provider(s) when I needed care
15.A health care provider made negative comments regarding my ethnicity, heritage or culture

The internal consistency reliability of the three measures was high with Cronbach’s alpha as follows: MADM (0.95), MOR (0.92) and MIST (0.85). For MADM, the mean score for the sample was 29.5 (range 7–42), for MOR it was 32.9 (range 7–42), and for MIST, it was 2.1 (range 0–15). Table 1 displays the MADM, MOR, and MIST items.

Respondents had the opportunity to provide open-ended comments describing the nature of their mistreatment. Some comments that explicate our quantitative findings are quoted in this paper.

### Data analysis

Chi-square testing examined differences in the proportion of respondents who scored in the top and bottom 33% of the MADM, MOR, and MIST across BMI categories with* p* < 0.05 used as an indicator of statistical significance (Table 3). Data were stratified using World Health Organization body mass index categories: underweight BMI < 18.5, normal weight BMI ≥ 18.5–24.9, overweight BMI ≥ 25–29.9, obesity class 1 BMI ≥ 30–34.9, and obesity classes 2 to 4, BMI ≥ 35 [37]. Individuals with underweight BMI were not included in this analysis as their health risks and management recommendations are distinct from other weight groups. Although BMI is an imperfect measure of adiposity and is not perfectly correlated with health, it is the measurement used in perinatal care and the research literature to quantify size and was used in this study [38].

For each of the three indices, the continuous scores were categorized into two distinct groups: 1) those with scores in the top third percentile and experiencing higher autonomy, respect, or mistreatment, labeled a “top 33%,” and 2) those scoring the bottom third percentile and experiencing a lower level of autonomy, respect, or mistreatment, labeled as “Bottom 33%.”

Separate analyses for the top and bottom tertiles yielded six regression models (Tables 4– 6). Multivariable logistic regression was used to calculate unadjusted and adjusted odds of key outcomes by BMI group, with normal weight (BMI range of 18.5–24.99) as the reference category; and adjusting for Indigenous or racialized identity (categorized as Indigenous or Person of Colour), education level, income sufficiency, and age. For the identity variable -Individuals identifying as Indigenous encompassed First Nations, Métis, or Inuk (Inuit), while People of Colour included Central Asian, East Asian, Latinx or Hispanic, Middle Eastern, South Asian, Southeast Asian, and Black individuals.

## Results

From July 2020 to January 2022, 6096 participants detailed their perinatal experiences through a mix of closed and open-ended questions. The current analysis included 3,280 participants after excluding individuals with a BMI under 18.5 and those with missing height or weight data. Table 2 shows that of these participants, approximately 53.2% had a pre-pregnancy BMI classified as normal, 26.4% were considered overweight, and one in five (20.4%) had a BMI of 30 or above (classes 1–4 obesity). Among the respondents, 5.7% self-identified as Indigenous, and 15.3% were from other racial groups, including Black, Latina, Asian, Middle Eastern, or a combination of race and/or ethnicities. In this paper, due to limitations in sample size, data from First Nations, Métis, and (Inuk)Inuit respondents were not stratified by distinct Indigenous groups. The remaining respondents self-identified as White (79.0%).

**Table 2:** Maternal Characteristics by BMI category

Weight Category	Normal BMI: 18.5–24.99 N (%)	Overweight BMI: 25–29.99 N (%)	Class 1 Obesity BMI: 30–34.99 N (%)	Class 2 and greater Obesity BMI > 35 N (%)
	1,744 (53.2%)	867 (26.4%)	386 (11.8%)	283 (8.6%)
**Racial Identity**				
Indigenous (a)	78 (4.5%)	54 (6.2%)	31 (8.0%)	25 (8.8%)
White	1,361 (78.0%)	689 (79.5%)	306 (79.3%)	235 (83.0%)
People of Colour (b)	305 (17.5%)	124 (14.3%)	49 (12.7%)	23 (8.1%)
**Income was Enough to Meet Needs**				
More Than Enough	999 (58.9%)	451 (53.6%)	182 (48.1%)	110 (39.6%)
Enough	596 (35.1%)	327 (38.9%)	162 (42.9%)	142 (51.1%)
Not Enough	101 (6.0%)	63 (7.5%)	34 (9.0%)	26 (9.4%)
**Education**				
> = High School	115 (6.8%)	64 (7.6%)	35 (9.3%)	29 (10.4%)
Some College/ Apprentice	320 (18.8%)	204 (24.2%)	111 (29.4%)	92 (33.1%)
Undergraduate	577 (34.0%)	293 (34.7%)	110 (29.1%)	102 (36.7%)
Graduate/Professional	687 (40.4%)	283 (33.5%)	122 (32.3%)	55 (19.8%)
**Age Categories**				
< 25	149 (8.6%)	85 (9.9%)	38 (9.9%)	33 (11.7%)
25–34	1,214 (70.1%)	570 (66.4%)	269 (70.2%)	175 (61.8%)
> 35	369 (21.3%)	204 (23.7%)	76 (19.8%)	75 (26.5%)

(a) First Nations, Métis, or Inuk (Inuit)

(b) includes Central Asian, East Asian, Latinx or Hispanic, Middle Eastern, South Asian, South East Asian, and Black

The sample was characterized by a high level of financial sufficiency, with 93.0% reporting “enough” or “more than enough” income to meet financial obligations and nearly 70% having attained an undergraduate, graduate, or professional degree. Most participants were aged over 25 at the time of pregnancy awareness, with 10% being under 25. Pre-pregnancy BMI was significantly associated with race/ethnicity, income sufficiency, and education but not with age (Table 2).

Several participants wrote clear comments about how weight stigma was the focal point of antenatal care, and how providers made assumptions based on weight alone.

*Despite being tall and somewhat overweight, every single appointment revolved around shaming me for my size. (Participant BMI 31.6).*

*A few doctors I saw made a point of mentioning my weight and went as far as telling me that I would likely not be able to birth vaginally as babies do not “come down” in obese women so I would likely need a cesarean. (Participant BMI 31.9)*.

One participant responded to the question, “If you could change one thing about your care during pregnancy, birth or after birth, what would that be?” with:

*Found my voice, not been paralyzed by worry of judgement related to my body size, not obsess that c section/struggle with breast feeding was a result of my bodies failure(s) due to its size. Implicit discrimination and judgement EVERYWHERE within the health care system…(Participant BMI 31.4).*

Although few participants wrote weight-stigma related comments, the three quotes above give specific voice to participants’ feelings of weight stigma which were quantified using the MADM, MOR, and MIST scores.

Table 3 illustrates that as BMI increased, the proportion of individuals reporting low respect and autonomy by MADM, MOR, and MIST scores tended to increase. For instance, the percentage of individuals reporting low autonomy in decision-making rose from 28.4% in the normal weight group to 39.1% in obesity classes 2 or greater. Similarly, mistreatment from healthcare providers was more prevalent among those with higher BMIs, with 40.6% of individuals in class 1 obesity reporting mistreatment compared to 26.6% in the normal weight range, reaching 42.0% in obesity classes 2 or greater.

**Table 3:** Prevalence of women in the bottom and top 33% of MADM, MOR and MIST scores across BMI categories

**Weight Category**	**Normal BMI: 18.5–24.99** **N (%)**	**Overweight BMI: 25–29.99** **N (%)**	**Class 1 Obesity** **BMI: 30–4.99** **N (%)**	**Class 2 and Greater Obesity BMI > 35** **N (%)**	Chi-Square Test* P value
Bottom 33% MADM score	481 (28.4%)	259 (31.1%)	127 (33.7%)	106 (39.1%)	0.002
Top 33% MADM score	715 (42.3%)	314 (37.7%)	140 (37.1%)	89 (32.8%)	0.006
Bottom 33% MOR score	405 (24.2%)	249 (30.0%)	122 (32.7%)	108 (39.4%)	< 0.001
Top 33% MOR score	707 (42.3%)	302 (36.4%)	119 (31.9%)	84 (30.7%)	< 0.001
Bottom 33% MIST score	594 (52.2%)	245 (42.0%)	118 (42.0%)	72 (36.0%)	< 0.001
Top 33% MIST score	302 (26.6%)	201 (34.5%)	114 (40.6%)	84 (42.0%)	< 0.001

*MADM *Mothers Autonomy in Decision Making Scale, *MORI *Mothers on Respect Index, *MIST* Mistreatment Index

Bottom 33% score: those with scores in the bottom third percentile of MADM, MOR, MIST and experiencing lower level of autonomy, respect, mistreatment

Top 33% score: those with scores in the top third percentile of MADM, MOR, MIST and experiencing higher level of autonomy, respect, mistreatment

Table 4 examines the association between BMI and autonomy in decision-making as measured by the Mother's Autonomy in Decision Making (MADM) scale. Individuals with BMIs in the overweight range obesity class 2 or higher had significantly higher odds of reporting lower MADM scores, indicating reduced autonomy. Specifically, individuals in obesity class 2 or higher showed notably higher odds of reduced autonomy, with an unadjusted odds ratio of 1.62 (62% higher likelihood) and an adjusted odds ratio of 1.45 (45% higher likelihood) compared to individuals with a normal BMI.

**Table 4:** Mothers Autonomy in Decision Making Scale (MADM) Odds Ratios by Body Mass Index and Select Demographics

	**Bottom 33% of MADM Score**		**Top 33% of MADM Score**	
Logistic Regression	**Unadjusted Odds Ratio** **(95% CI)**	**Adjusted Odds Ratio** **(95% CI)**	**Unadjusted Odds Ratio** **(95% CI)**	**Adjusted Odds Ratio** **(95% CI)**
**BMI Category**				
18.5–24.99	1	1	1	1
25–29.99	1.14 (0.95, 1.36)	1.11 (0.92, 1.34)	0.83* (0.70, 0.98)	0.83* (0.70, 0.99)
30–34.99	1.28* (1.01, 1.62)	1.17 (0.91, 1.50)	0.81 (0.64, 1.02)	0.86 (0.68, 1.09)
≥ 35	1.62** (1.24, 2.11)	1.45** (1.10, 1.91)	0.67** (0.51, 0.88)	0.71* (0.54, 0.94)
**Racial Identity**				
White		1		1
Indigenous (a)		1.47* (1.07, 2.03)		0.83 (0.59, 1.16)
People of Colour (b)		1.32* (1.06, 1.65)		0.75** (0.60, 0.92)
**Income Enough to Meet Needs**				
More Than Enough		1		1
Enough		1.52** (1.28, 1.80)		0.75** (0.64, 0.88)
Not Enough		2.11** (1.55, 2.88)		0.47** (0.34, 0.67)
**Education Category**				
< = High School		1		1
Some College/Apprentice		0.76 (0.55, 1.03)		1.29 (0.92, 1.81)
Undergraduate		0.67* (0.49, 0.92)		1.27 (0.91, 1.78)
Graduate/Professional		0.59** (0.43, 0.81)		1.47* (1.04, 2.06)
**Age Category**				
< 25		1		1
25–34		0.85 (0.65, 1.12)		1.39* (1.03, 1.87)
> 35		0.68* (0.49, 0.93)		1.48* (1.06, 2.07)
Number of Observations	3171	3068	3171	3068

^**^ *p* < .01. ^*^ *p* < .05. 95% Confidence Interval (CI). Robust estimators are used

Bottom 33% MADM score: those with scores in the bottom third percentile and experiencing lower level of Autonomy

Top 33% MADM score: those with scores in the top third percentile and experiencing higher level of Autonomy

(a) First Nations, Métis, or Inuk (Inuit); (b) People of Colour include Central Asian, East Asian, Latinx or Hispanic, Middle Eastern, South Asian, Southeast Asian, and Black

**Fig. 1:**
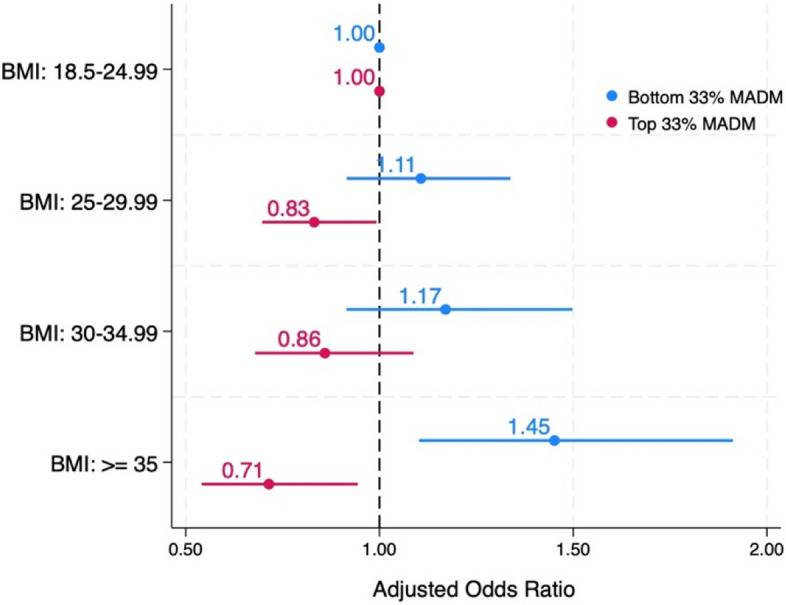
Adjusted Odds Ratios for Mothers Autonomy in Decision Making (MADM) Scores by Body Mass Index Adjusted for: Race/ethnicity, Income, Education, Age. The lines represent the 95% confidence interval levels. Bottom 33% MADM score: those with scores in the bottom third percentile and experiencing lower level of Autonomy. Top 33% MADM score: those with scores in the top third percentile and experiencing higher level of Autonomy

Individuals with higher BMIs consistently reported lower autonomy scores. In the adjusted model, individuals classified as overweight were less likely to report high autonomy, with adjusted odds ratio (AOR) of 0.83. Those in obesity class 2 or higher displayed an even lower likelihood of attaining the highest tier of autonomy scores, with an adjusted odds ratio of 0.71. Figure 1 illustrates the pattern showing that increasing BMI is associated with decreasing levels of high respectful care and increasing levels of low respectful care; however, only the scores of those with BMIs > 35 reached statistical significance.

**Fig. 2:**
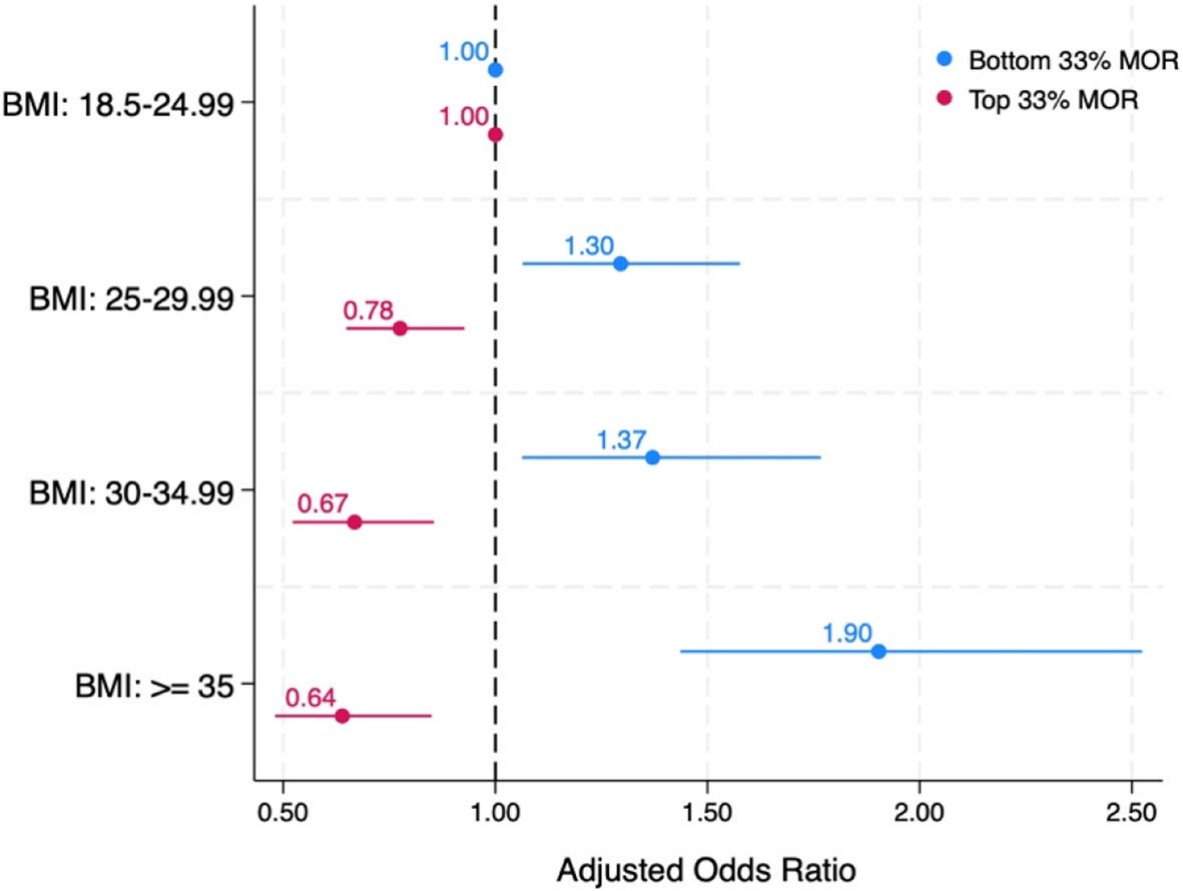
Adjusted Odds ratios for Mothers on Respect (MORI) by Body Mass Index Categories Adjusted for: Race/ethnicity, Income, Education, Age. The lines represent the 95% confidence interval levels. Bottom 33% MORI score: those with scores in the bottom third percentile and experiencing lower level of respectful care. Top 33% MORI score: those with scores in the top third percentile and experiencing higher level of respectful care

This research also uncovered significant relationships between BMI categories and MOR scores (Table 5). As BMI increased, so did the odds of reporting lower levels of respectful care. Individuals classified as overweight, obesity class 1, or obesity class 2 or higher exhibited adjusted odds ratios of 1.30, 1.37, and 1.9, respectively, with all being statistically significant (*p* < 0.01). Conversely, individuals in these BMI categories had reduced odds of being in the highest tertile of MOR scores with odds ratio of 0.78, 0.67, 0.64 (all *p* < 0.01) (Fig. 2).

**Table 5:** Mothers on Respect Index (MORI) Odds Ratios by Body Mass Index and Select Demographics

	**Bottom 33% of MORI Score**		**Top 33% of MORI Score**	
Logistic Regression	**Unadjusted Odds Ratio** **(95% CI)**	**Adjusted Odds Ratio** **(95% CI)**	**Unadjusted Odds Ratio** **(95% CI)**	**Adjusted Odds Ratio** **(95% CI)**
**BMI Category**				
18.5–24.99	1	1	1	1
25–29.99	1.34** (1.11, 1.62)	1.3** (1.06, 1.58)	0.78** (0.66, 0.93)	0.78** (0.65, 0.93)
30–34.99	1.52** (1.19, 1.94)	1.37* (1.06, 1.77)	0.64** (0.50, 0.81)	0.67** (0.52, 0.85)
≥ 35	2.03** (1.56, 2.66)	1.9** (1.44, 2.52)	0.6** (0.46, 0.79)	0.64** (0.48, 0.85)
**Racial Identity**				
White		1		1
Indigenous (a)		1.75** (1.27, 2.42)		0.65* (0.45, 0.93)
People of Colour (b)		1.35** (1.08, 1.70)		0.72** (0.58, 0.89)
**Income Enough to Meet Needs**				
More Than Enough		1		1
Enough		1.51** (1.26, 1.81)		0.7** (0.59, 0.82)
Not Enough		2.8** (2.04, 3.83)		0.43** (0.30, 0.62)
**Education Category**				
< = High School		1		1
Some College/Apprentice		1.1 (0.80, 1.51)		1.21 (0.86, 1.70)
Undergraduate		0.81 (0.59, 1.12)		1.2 (0.85, 1.68)
Graduate/Professional		0.84 (0.60, 1.18)		1.24 (0.88, 1.75)
**Age Category**				
< 25		1		1
25–34		0.7* (0.53, 0.93)		1.47* (1.08, 2.00)
> 35		0.52** (0.37, 0.72)		1.68** (1.19, 2.36)
Number of Observations	3147	3046	3147	3046

^**^p < .01. ^*^ *p* < .05. 95% Confidence Interval (CI). Robust estimators are used

Bottom 33% MORI score: those with scores in the bottom third percentile and experiencing lower level of respectful care

Top 33% MORI score: those with scores in the top third percentile and experiencing higher level of respectful care

(a) First Nations, Métis, or Inuk (Inuit); (b) People of Colour include Central Asian, East Asian, Latinx or Hispanic, Middle Eastern, South Asian, Southeast Asian, and Black

**Fig. 3:**
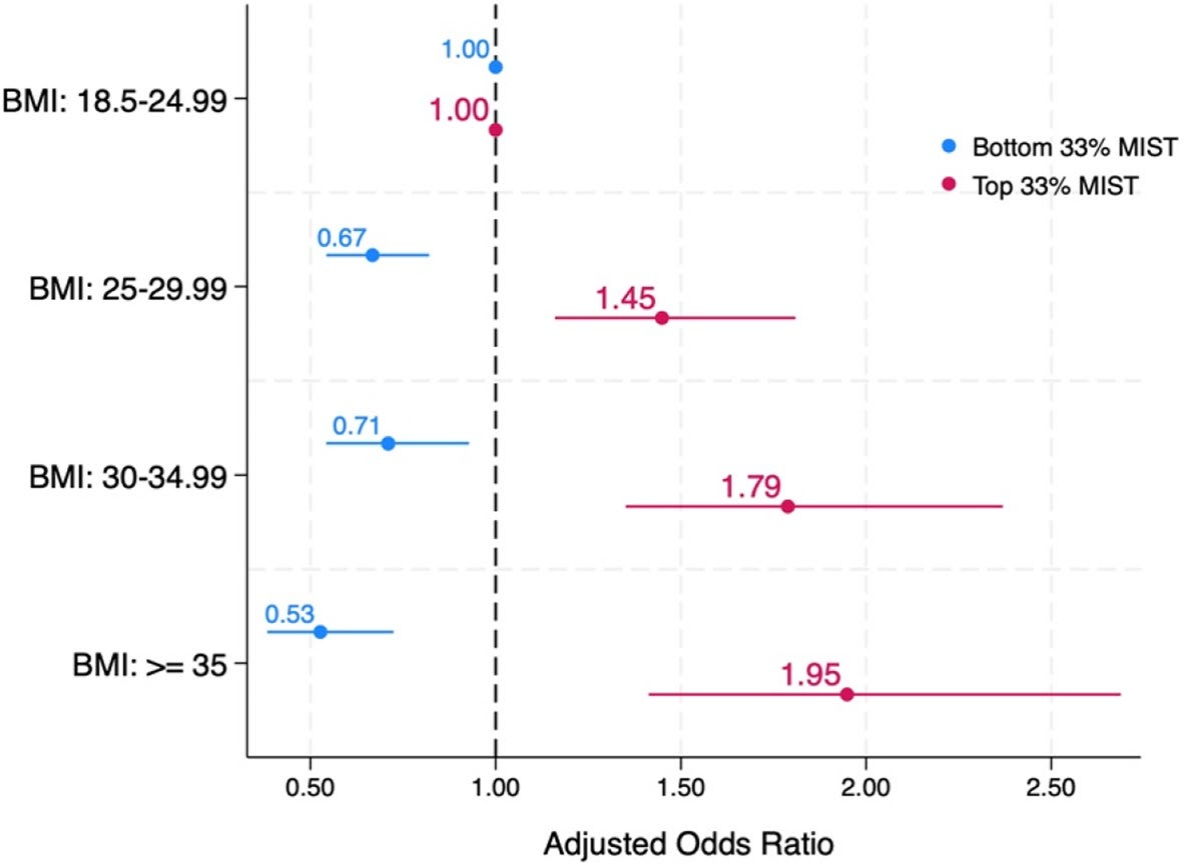
Adjusted Odds Ratios for Mistreatment Index (MIST) by Body Mass Index Categories Adjusted for: Race/ethnicity, Income, Education, Age. The lines represent the 95% confidence interval levels. Bottom 33% MIST score: those with scores in the bottom third percentile and experiencing lower level of Mistreatment. Top 33% MIST score: those with scores in the top third percentile and experiencing higher level of Mistreatment

Table 6 illustrates a significant association between BMI categories and the likelihood of mistreatment during perinatal care. The adjusted odds ratios indicate a consistent pattern of increased mistreatment among individuals with higher BMIs. Specifically, the adjusted odds of having scores in the top 33% of the Mistreatment Index (MIST) for individuals classified as overweight, obesity class 1, and obesity class 2 or higher were 1.45, 1.79, and 1.95, respectively, all statistically significant at *p* < 0.01. In other words, individuals with a pre-pregnancy BMI ≥ 35 were nearly twice as likely to report mistreatment from providers, even after accounting for other socio-demographic factors. Additionally, those classified as overweight, obesity class 1, and obesity class 2 or higher had reduced odds of being in the lowest tertile of MIST scores (indicating less mistreatment), with odds ratios of 0.67, 0.71, and 0.53, all statistically significant at *p* < 0.01 (Fig. 3).

**Table 6:** Mistreatment Index (MIST) Odds Ratios by Body Mass Index Categories and Select Demographics

	**Bottom 33% of MIST Score**		**Top 33% of MIST Score**	
**Logistic Regression**	**Unadjusted Odds Ratio** **(95% CI)**	**Adjusted Odds Ratio** **(95% CI)**	**Unadjusted Odds Ratio** **(95% CI)**	**Adjusted Odds Ratio** **(95% CI)**
**BMI Category**				
18.5–24.99	1	1	1	1
25–29.99	0.66** (0.54, 0.81)	0.67** (0.54, 0.82)	1.45** (1.17, 1.80)	1.45** (1.16, 1.81)
30–34.99	0.66** (0.51, 0.86)	0.71* (0.54, 0.93)	1.89** (1.44, 2.48)	1.79** (1.35, 2.37)
≥ 35	0.51** (0.38, 0.70)	0.53** (0.38, 0.72)	2.0** (1.47, 2.73)	1.95** (1.41, 2.69)
**Racial Identity**				
White		1		1
Indigenous (a)		0.47** (0.30, 0.72)		2.26** (1.53, 3.33)
People of Colour (b)		0.95 (0.73, 1.23)		1.33* (1.02, 1.75)
**Income Enough to Meet Needs**				
More Than Enough		1		1
Enough		0.75** (0.62, 0.91)		1.64** (1.34, 2.01)
Not Enough		0.41** (0.27, 0.60)		2.56** (1.76, 3.71)
**Education Category**				
< = High School		1		1
Some College/Apprentice		0.74 (0.50, 1.09)		1.05 (0.70, 1.55)
Undergraduate		0.78 (0.53, 1.14)		1.12 (0.75, 1.66)
Graduate/Professional		0.74 (0.50, 1.09)		1.09 (0.73, 1.64)
**Age Category**				
< 25		1		1
25–34		1.36 (0.97, 1.90)		0.82 (0.58, 1.14)
> 35		1.66** (1.14, 2.41)		0.63* (0.43, 0.93)
Number of Observations	2201	2188	2201	2188

^**^ *p* < .01. ^*^ *p* < .05. 95% Confidence Interval (CI). Robust estimators are used

Bottom 33% MIST score: those with scores in the bottom third percentile and experiencing lower level of Mistreatment

Top 33% MIST score: those with scores in the top third percentile and experiencing higher level of Mistreatment

(a) First Nations, Métis, or Inuk (Inuit); (b) People of Colour include Central Asian, East Asian, Latinx or Hispanic, Middle Eastern, South Asian, Southeast Asian, and Black

The results also emphasize the significance of considering factors such as race, income sufficiency, and age in understanding perinatal care experiences. For example, multivariate logistic analysis revealed that individuals identifying as Indigenous or as a Person of Colour were more likely to report lower levels of autonomy (AOR 1.47 and 1.32, respectively, Table 4), less likely to report respectful care (AOR 0.65 and 0.72, respectively, Table 5), and more likely to report mistreatment (AOR 2.26 and 1.33, respectively, Table 6). Furthermore, better perinatal care experiences were reported by individuals in higher age categories across all three domains.

The analysis also demonstrated that individuals with higher income sufficiency reported better care experiences: the likelihood of reporting top tertial MADM (Table 4) and MOR (Table 5) scores decreased for those with "Enough Income" (AOR 0.75 and 0.70, respectively) and decreased further for those with "Not Enough Income" (AOR 0.47 and 0.43, respectively). A similar pattern was observed for MIST scores, where the likelihood of lower levels of mistreatment was reduced for those with "Enough Income" and "Not Enough Income" (AOR 0.75 and 0.41, respectively, in Table 6).

## Discussion

This large Canadian study with more than 3,000 participants demonstrated that pre-pregnancy BMI was significantly associated with race/ethnicity, income sufficiency, and education but not with age. Individuals with higher BMIs were more likely to experience income insufficiency, have lower levels of education, and more frequently self-identified as Indigenous or White. Those with BMI ≥ 35 exhibited notably higher odds of reduced autonomy (MADM) scores, with an unadjusted odds ratio of 1.62 (62% higher likelihood) and an adjusted odds ratio of 1.45 (45% higher likelihood) compared to individuals with a normal weight. Individuals with BMI of 25–25.9, 30–34.9, and ≥ 35 exhibited odds of falling into the lower tertile of respect (MOR) scores of 1.34, 1.51, and 2.04, respectively (*p* < 0.01). The odds of reporting higher rates of mistreatment (top 33% MIST scores) increased as BMI increased. Sociodemographic factors, such as race and income, affect mistreatment during perinatal care, but controlling for these underscores that BMI alone can play a significant role in shaping harmful experiences.

Those in the highest BMI category, class 2 obesity or higher, (BMI ≥ 35) had a 45% increased likelihood of being in the bottom tier of autonomy scores compared to individuals with a normal BMI range. This pattern suggests a concerning association where higher levels of BMI are consistently linked with lower reported autonomy. This lack of autonomy could represent judgements by antenatal care providers that people with obesity do not care for themselves and thus are neither capable nor deserving of decision making authority [7]. Studies have documented that participants thought their providers were disgusted by their fat and put them in a box, that is imposed routine recommendations to reduce risk instead of taking a holistic view of the person’s health, and advised testing, such as sleep apnea testing, based solely on BMI [26, 39].

Standardized obesity management guidelines [9–13] can be applied to a care plan, often without engaging the pregnant person in decision-making around options or recommendations for care [7, 26, 39].

Antenatal providers may also retreat to standardized management plans when feeling insufficiently prepared to discuss obesity in pregnancy. Swedish researchers surveyed 274 perinatal care providers (75% midwives, 25% obstetricians) about their attitudes toward obesity in pregnancy and administered three surveys to measure obesity related bias [40]. One-third of participants found talking about obesity to be more sensitive than talking about smoking or alcohol use. Fear of making the women ashamed or worried kept 17% of the midwives from discussing weight during pregnancy [40]. This fear of weight shaming, lack of clinic time, and insufficient educational resources was found in other studies of antenatal care providers [30, 41–44]. Loss of autonomy may also result from fetal focused medicine, where the needs of the fetus are made more important than that of the pregnant person [44]. Pregnant people experience weight stigma when the potential risk for the fetus is attributed to obesity, thus the pregnant person is blamed for potentially poor outcomes [44].

This study found that the odds of falling into the bottom 33% of MOR scores increased as BMI categories moved from underweight to class 2 obesity or higher. In addition, individuals with BMI ≥ 30 had reduced odds of being in the top 33% of MOR scores. These results suggest that BMI plays a significant role in shaping the patient-reported experience of maternity care, with people with higher BMIs, constructed as overweight and obesity, having adverse experiences, possibly influenced by weight stigma in maternity care settings. The findings underscore the importance of considering perinatal care practices related to high weights to ensure respectful and positive experiences for all individuals, regardless of their weight status.

The results from the logistic regression analysis highlight a clear association between various BMI categories and the likelihood of experiencing mistreatment during perinatal care. A combination of sociodemographic factors, including race and income, influences mistreatment during perinatal care. Even after controlling for these, BMI played a significant role in shaping experiences during pregnancy and the postpartum. The intersectional stigma of race, low income, and weight heightens the potential for disrespect during care. This stigma adds stress to pregnant peoples’ experience, exacerbating the potential for disordered eating and excessive gestational weight gain [45].

Implicit and explicit bias in health care providers has been documented [2, 29, 40, 43]. Race is associated with obesity with Indigenous and People of Colour more likely to have weights in the obesity ranges [17, 46]. Indigenous pregnant people face heightened stigma and maltreatment from healthcare providers, not only due their higher BMIs but by systemic biases ingrained within the healthcare system and the persistent influence of colonial health policies [47, 48]. Discrimination acts to further widen disparities in perinatal healthcare outcomes among Indigenous populations [47]. Health care providers, researchers, and policymakers alike must take into consideration the ways in which social determinants of health have a synergistic impact on all Indigenous pregnant people, and not just First Nations individuals living in urban centres [49]. Health professionals have urged taking a weight neutral approach that integrates traditional Indigenous knowledge with a Health at Every Size® approach [50]. Focusing on health rather than simply combatting disease can increase body sovereignty among the Indigenous [50].

The Health at Every Size® (HES) approach has been recommended to reduce weight bias in perinatal care, citing the incorrect use of BMI as a proxy for overall health [51, 52]. HES shifts the focus away from weight to health, which requires clinicians to self-evaluate their weight biases and assure that they use weight inclusive language. A practice must have a welcoming environment for pregnant people of all sizes. Additionally, clinicians must recognize the formulaic approach of many treatment guidelines, and tailor care for those with high BMIs based on overall health following informed choice discussions [51, 52].

For perinatal care clinicians to provide more compassionate care, they will need an understanding of excess weight as an intergenerational physical and epigenetic adaptation to multiple socioeconomic disparities, including the nonnutritive diets available to people with insufficient income, chronic stressors such as racism, and exposure to unsafe, polluted neighbourhoods [46]. A 2024 scoping review of evaluated interventions to improve respectful maternity care found only 10 reports with 8 being done in Africa, one in Mexico and one in the United States [27]. The most common approach was provider training in respectful care. Wall posters about respectful care, provider counseling, constant feedback from patients, and improvements in equipment and infrastructure to reduce provider and patient frustration during care were also used. All interventions reduced disrespectful care and mistreatment based on patient feedback and post-intervention testing [27]. Group work that exposes clinicians’ implicit and explicit biases, followed by education regarding the social determinants of high weights, has the potential to increase clinician compassion and reduce microaggressions and mistreatment during perinatal care.

### Strengths and limitations

The study is unique in its use of quantitative patient-reported indices (MADM, MOR, MIST] to assess the quality of perinatal care. These indices enable a standardized evaluation of different aspects of respectful care, specifically focusing on autonomy, respect, and mistreatment. The more than 3,000 participant sample size presents a group diverse in race, ethnicity, and body size. With a 20% obesity rate, the sample neither under-represents nor over-represents people living with obesity.

As with all survey data, the similarity between survey respondents and those who did not respond is unknown. Pregnant peoples’ memories may not reflect the context of care decisions or interactions. Many respondents did not provide weight data. If these omissions formed a pattern is unknown. For example, if individuals in the higher obesity category were less likely to report weight, the results would be biased downwards. Conversely, if people with higher weights who had experienced mistreatment were more likely to complete the survey, reports of mistreatment might be inflated. Continued research using the MADM and MOR surveys and the MIST index will increase knowledge of weight stigma in perinatal care.

## Conclusion

This study provides a compelling illustration of pervasive weight stigma in Canadian healthcare that increases with higher weights. Weight, as quantified by BMI, plays a significant role in shaping perinatal care experiences with individuals facing notable weight stigma, disrespect, and mistreatment. These findings call for a critical evaluation of current healthcare practices and the implementation of comprehensive strategies to ensure equitable, respectful care for all, regardless of body weight. Professionals must identify their own implicit and explicit weight bias and seek education in the multiple social determinants of health that contribute to excess weight and culturally safe care. This may enable those professionals to reflect on their own biases and then to provide more respectful, less stigmatizing care, thereby improving perinatal care for people with high weights.

## Data Availability

The data that support the findings of this study are maintained by the Birth Place Lab, Midwifery, Department of Family Practice, University of British Columbia. Questions about data availability and materials can be addressed to https://www.birthplacelab.org/contact-us/.
